# The Relationship between Teachers and Pupils with Down Syndrome: A Qualitative Study in Primary Schools

**DOI:** 10.3390/bs13030274

**Published:** 2023-03-20

**Authors:** Agostino Carbone, Michela Castaldi, Giordana Szpunar

**Affiliations:** 1 Department of Developmental and Social Psychology, Faculty of Medicine and Psychology, Sapienza University of Rome, 00185 Rome, Italy; 2 Ministry of Education and Merit, 00153 Rome, Italy

**Keywords:** learning process, inclusion, disability, primary school, teachers, psychological intervention for school, grounded theory

## Abstract

Down syndrome (DS), due the presence of an intellectual disability and a precarious health condition, involves important challenges for developing skills at school. The study explores the relational dynamics between teachers and students with DS and how the latter influence the processes of socialization and learning at school. The study involved 15 (*n* = 15) special education teachers (*M _age_* = 40.4; *SD* = 9.3) from primary schools in Italy who were or had previously been in charge of a pupil with DS. The teachers were interviewed through a semi-structured interview, built ad hoc. The data were analyzed through the grounded theory method supported by ATLAS.ti software. Data analysis produced 20 (*n* = 20) categories grouped into 6 (*n* = 6) macro-categories: (1) *psychophysical characteristics*; (2) *learnings*; (3) *relational aspects*; (4) *collaborations*; (5) *extracurricular activities;* and (6) *teacher training.* The research shows that students with DS have good relationships with their peers; however, there are still some important critical issues, including poor training of teachers on certain topics and on the application of collaborative and relationship support strategies, the limited collaboration between support teachers and curricular teachers, and the absence of support from ad hoc professional figures.

## 1. Introduction

### 1.1. Down Syndrome

Down syndrome (DS) is one of the most studied genetic syndromes [1]. It affects both sexes and in the general population affects 1:700–1:1000 births with a prevalence of 1:2000 [2]. According to the Centers for Disease Control and Prevention [3], DS is the most common chromosomal condition, affecting approximately 6000 children in the United States each year. In Europe, are estimated that for every 10,000 births, 11,2 infants are affected by Down syndrome [1,4]. As far as Italy is concerned, according to data reported in 2020 by the “Down Syndrome” Center of the “Bambin Gesù” Pediatric Hospital, DS affects approximately 38,000 people and, therefore, 1 child in every 1200 born. From the estimates made by the Italian Association of people with Down Syndrome (AIPD), between 38,000 and 40,000 individuals with this syndrome live in Italy, and approximately 61% of them are over 25 years old. However, considering that there are many voluntary abortions (following prenatal diagnosis) and that 78% of pregnancies with fetuses affected by trisomy 21 terminate spontaneously, the conception of children with DS is higher than those born [4].

DS affects all ethnic groups and has very ancient roots. In Greece, the remains of a person from the Neolithic era have been found with characteristics that could be compatible with DS [5].

People with this syndrome have 47 chromosomes instead of 46 and, for this reason, the cause of this syndrome is genetic [4]. As Davis [6] and Cottini [4] point out, three different forms of trisomy 21 have been identified, in which an extra chromosome 21 is always present; however, it can be free, translocated to another chromosome, or affect only some cells.

The cause of the presence of an extra chromosome in position 21 in subjects with DS is not yet known, but a correlation with the age of the mother is evident. Analyzing the data, it appears that out of 2000 children born to mothers under the age of 20, Down syndrome is found in less than one case. Instead, DS was found in 20–60 children of women over the age of 45 [4].

### 1.2. Clinical, Relational, and Cognitive Aspects and Comorbidities

Individuals with DS have particular characteristics from birth concerning the hypotonia of the muscles, the joints, and the head. Furthermore, trisomy 21 is frequently associated with various types of health problems and pathologies, such as congenital heart disease in 50% of cases, visual impairment (such as strabismus, congenital cataract, and myopia) in 50–70% of cases, auditory impairments (which in some cases further aggravates the linguistic evolution) in 50–70% of cases, epileptic seizures (specifically infantile spasms) in 5% of cases, autoimmune pathologies (such as celiac disease and diabetes), thyroid diseases (in particularly subclinical hypothyroidism is present in 25–305 of the cases), orthopedic problems, breathing problems during sleep (due to weak muscles and poor muscle tone), blood disorders (anemia and low platelet counts), gastrointestinal diseases, urinary tract and kidney defects, and premature aging. Another typical feature of people with DS is the delay in height growth [6,7]. Furthermore, as happens with other forms of intellectual disability, psychological depressive/behavioral problems are also frequent in subjects with DS. It is estimated that in people with intellectual disabilities that compared with the general population, comorbidity with other mental disorders is between three and four times higher.

Among the externalizing disorders manifested by individuals with DS are impulsiveness, opposition and attentional problems [8], and obsessive-compulsive disorder. The latter will be “[…] characterized by repetitive behaviors with a search for gratification such as food compulsiveness, polydipsia, hyperventilation” [2]. Furthermore, as Shaw points out, children with DS are also frequently diagnosed with ADHD, which is estimated to affect approximately 6–8% of subjects [2].

Subsequently, towards adolescence we witness a different clinical picture, with a decrease in externalizing disorders and an increase in internalizing disorders, such as anxiety and depression (characterized by fatigue, psychomotor retardation, sleep disorders, obesity and feelings of guilt) [2]. Their cognitive level varies, a characteristic known as “high internal variability” of the syndrome, and which must be taken into consideration before planning any type of intervention, including educational interventions [9]. However, in most individuals with DS, the level of cognitive functioning varies between moderate and severe, the mean IQ is 50, and the adaptive level is in line with mental age [2]. Overall, the neuropsychological profile of children with DS, in addition to being characterized by intellectual disability, presents developmental unevenness between the various cognitive skills. The global alteration of processes in people with DS involves motor, linguistic, and cognitive development, self-care skills, and personal and social dimensions [1,10].

In general, the delay in motor development and the impairment of verbal language often add to intellectual disability, and this frequently implies difficulties that lead the person to the acquisition of autonomy [9]. In addition to these difficulties related to the condition of DS itself, the growth towards autonomy is often also hindered by the fearful attitudes of adults (especially parents) and by the ambivalences of the environment, as these elements interfere with the individual’s degree of potential autonomy. Contardi [11] states that today, 13% of adults with DS work (although there could be many more); in 1979, there were no workers with this syndrome. In these forty years, other factors have changed alongside employment for those with DS. The quality of life for individuals with DS has greatly improved, as has their survival. There have been numerous advances in the medical field that have made it possible to treat pathologies very often associated with DS, including primary causes of death for people with this syndrome. Specifically, these include treatments for heart disease, diseases affecting the thyroid, and leukemia. These evolutions in the field of medicine have led to a substantial increase in the life expectancy of people with DS. In Italy, this has increased from an average of 33 years to approximately 62 years [12].

After early adulthood, people with DS often go through a phase of decline from a cognitive point of view, with the manifestation of dementia [2]. Carr and Collins [13] conducted a longitudinal study on a group of 54 individuals with DS with the intention of following them from infancy to age 50; however, of the initial 54 subjects, they were able to follow only 27 until the end. Analyzing the results of the tests shows a significant drop in memory tests, the non-verbal intellectual test (which evaluates graphic, spatial, constructive skills, etc.) and in self-help. A very different situation appears for the verbal tests in which few changes emerged. Additionally, 5 (18%) of 27 people with DS reviewed at age 50 exhibited signs of dementia [13]. In general, it is estimated that approximately 36–66% of individuals with DS aged 50 to 59 are diagnosed with Alzheimer’s [2]. Such a high percentage of this diagnosis compared with the general population lies in neuropathological and neurochemical correlations that have been identified between DS and Alzheimer’s [2].

### 1.3. Perceptions of Teachers

Several studies have demonstrated the existence of correlated factors that influence the success of the process of inclusion of children with DS; they concern the perceptions of the teacher towards children with disabilities, the available resources necessary for the realization of the inclusive program, and knowledge on the school background. The first of these three factors was examined by a systematic review of 11 studies conducted globally between 2000 and 2018 [14]. Specifically, this review shows that the positive perceptions and behaviors of teachers towards inclusive education programs aimed at children with DS play a significant role in the success of the program itself. Furthermore, it appears that when developing the individual educational program, teachers are more willing to support the benefits of inclusion when they believe and trust in the success that will be achieved by children with DS in their class group. The authors of a study aimed at analyzing teachers’ perceptions [15] of the successful transition from kindergarten to primary school for children with and without DS state that the former may need support and adjustments; however, if initial expectations of the teacher are lower for children with DS than for children without disabilities, then it is probable that the gap between the two groups, which emerged at school entrance, will increase more and more over time. In the same study, the factors that they consider most important to allow children with DS to achieve good performance, concern aspects related to how much the child is willing to go to school and how much they manage to adapt within the classroom. The review also shows how much the cultural aspect influences the ideas of teachers. Specifically, it shows that in the United Kingdom compared to the United Arab Emirates, there are higher expectations for children with DS. Therefore, it is important to investigate and increase teachers’ expectations and perceptions because they influence the organization of the transition from kindergarten to primary school, and in general all work to be carried out by pupils.

Among the behaviors and attitudes of teachers that strongly influence the learning process of students and their results, according to Hattie and Timperley [16], there is both negative and positive feedback. Feedback can be defined as feedback that a subject receives as a result of what has been accomplished, and it is effective if it makes the student understand their point of arrival, reminds them of the set goal, and shows them the next step to take to achieve it. Schwab, Huber, and Gebhardt [17] conducted a study aimed at investigating the influence of teachers’ feedback on the social acceptance of students with DS. They noted that the way and frequency with which a teacher scolds, or conversely praises and encourages, a child with DS causes an impact on the type of relationship that will be established between the pupil with the disability and his/her classmates. This experimental study concluded that the child with DS is accepted within their class group to a lesser extent than the child without disabilities. To overcome this problem, the authors underline the importance of developing inclusive programs focusing on the interaction between the teacher and the pupils of the class, on the training of teachers on the most effective behaviors, and on how to provide feedback appropriately. For example, studies by Campbell, Glimore, and Cukelly [18] have shown that training characterized by a theoretical part and structured experiences carried out in the field can change the attitude of the teacher towards students with Down syndrome. In fact, at the end of a six-month training period focused on school inclusion and on information related to atypical development and disability, undergraduate students of primary education at an Australian university have shown to have acquired a more accurate knowledge of DS, to have developed more positive attitudes towards inclusive education of children with Down syndrome, and have changed their attitude towards disability in general, reporting greater ease in interacting with people with disabilities. These results suggest that training teachers on a specific disability (in this case DS) instigates changes in teachers’ attitudes towards disability. By analyzing the data obtained from the questionnaires submitted to the students before the start of the training course, it appears that they had stereotyped opinions about individuals with DS. Most of them said that children with DS were more affectionate and friendly, a small proportion that they were more aggressive, and half said that they were more vulnerable. The effectiveness of training to avoid running into these problems for pupils with DS is highlighted by numerous studies; in particular, Batanero et al. [19] show the existence of a positive correlation between teacher education (about DS) and their years of experience with children with DS on the one hand, and their positive perceptions about the inclusion of pupils with DS on the other. Aware of this correlation, there are many teachers who explicitly ask for greater training both to improve the teaching process aimed at pupils with DS, learning from their potential, and to be able to include such pupils within the class group. In particular, teachers who have little experience with children with DS say they want specific training because they feel anxious when they do not feel ready to meet the specific needs of these pupils. Furthermore, they stated that they are aware of the potential that training brings regarding the change of their perceptions and negative ideas about the process of inclusion of children with disabilities and regarding the development of positive attitudes towards inclusive programs. The importance of training in the school environment is also highlighted by John Hattie in the book *Visible Learning* [20] where he states that the initial and continuous training and professional development of teachers are the main aspects that the effectiveness of education systems. He believes that “the evolutionary path of the students passes above all through the teachers, […] and acting on their quality translates into acting indirectly on the quality of the results achieved” ([21] p. 4).

## 2. Current Research

Since the 1970s, Italy has taken a unique approach towards special education for children with intellectual and other disabilities. All students with intellectual disabilities in Italy are educated in typical classrooms alongside their peers without disabilities, rather than in special education classes as is often the case in many other countries. Following the inclusion of students with disabilities in normal classes, a new professional figure was established in Italy in the field of teaching: the support teacher. Their task is to develop the relational skills of students with disabilities, support learning, promote a culture of inclusion in the school and in the classroom, and act as a relational facilitator between the pupils and the curricula teachers.

In order to contribute to a better professional definition of the function and tasks of the support teacher, research was conducted on the relationship between support teachers and a particular group of students with a specific and common disability present in the school: Down syndrome. The aim of the research was to collect and analyze the experiences of support teachers with pupils with DS in primary school.

The specific objectives of the research are: (1) to collect information on the emotions that teachers experience within the relational process that accompanies the support and development of a student with disabilities within a time frame of at least one school year; (2) consider the critical issues that DS entails for pupils in the process of acquiring autonomy; and (3) highlight the coping strategies that teachers use when solving learning and relationship problems.

## 3. Methodology

### 3.1. Participants

The group of participants (see Table 1) consisted of 15 (*n* = 15) primary special education teachers (*M_age_* = 40.4; *SD* = 9.3) who are caring for, or were caring for, a pupil with DS. The inclusion criteria included: participants who, at the time of research, were support teachers in a primary school, or had been in the last 3 years, and those who had directly assisted a pupil with DS. The participants, all female, were identified through telephone contact. With the exception of 2 teachers who worked in medium-large cities in northern Italy, all others taught in schools in central and southern Italy. Specifically, almost half (7 of them) taught in schools in Campania, 2 in Puglia, 1 in Molise, another in Abruzzo, and 2 in Rome. They ranged in age from 25 to 54. Of the 15 teachers interviewed, 8 were tenured, 1 was in her probationary year, and the remaining 6 were untenured. The average years of service in the support post was 10.8. Considering both the years of teaching in the general position and in the support position, the average of the years of service of the entire group was 13.06.

Furthermore, 12 teachers were qualified to support, and 2 of them were attending the Italian 1-year training program for teaching people with special educational needs and disabilities [“Tirocinio Formativo Attivo Sostegno” (T.F.A.)]. Only 1 support teacher, being still enrolled in the university course of primary education, was employed under an apprenticeship contract.

### 3.2. Procedures

The development of the research design took place in several phases. On the basis of scientific literature, the thematic areas to be investigated were identified and then the specific questions for the participants were formulated. The interviews were conducted between March 2022 and May 2022, had a duration of 40 to 50 min, and were conducted remotely and audio recorded. Participants were asked to sign an informed consent, which was then acquired electronically by the principal investigator. The participants were reassured about the confidential treatment of the data. The research design, the administration procedures, the collection, and custody of the data were approved by the Ethics Committee of Sapienza University of Rome (protocol code 0000687). The research group was made up of 2 senior researchers and a junior researcher, all with teaching experience in Italian schools. The interviews were conducted by the junior researcher.

A subsequent phase of the research envisaged the integral transcription of the data that were collected in a confidential form. Finally, we proceeded with the analysis of the textual material and with the definition of the results.

### 3.3. Instrument

A semi-structured interview [22] was constructed consisting of 18 guiding questions and the interview guide (Appendix A) was developed through the study of the salient literature on the subject and from the direct experience of the research group in the school context. The theoretical framework was inspired the demand analysis model [23] in psychology, a psychodynamic model oriented towards the analysis of relationships within institutional and group settings. When it was deemed necessary, the interviewer could also ask other questions to receive clarifications and to unravel doubts about what the interviewee said. In some circumstances, the order of the questions has been changed depending on the answers received. The interviewee was left free to express themselves during the entire verbal exchange. The thematic areas that have been investigated can be traced back to the following dimensions: student characteristics; relational aspects and inclusion; methodological decision-making dimensions; out-of-school environment; teacher’s experiences and reflections; relationship between school and family.

## 4. Data Analysis

After collecting the interviews, they were transcribed verbatim and analyzed using a qualitative method of an interpretative type, specifically grounded theory [22]. The analysis was supported by ATLAS.ti software [24]. The methodology of grounded theory allows the researcher to highlight the categories of analysis from the available data without developing them from the literature review; the latter is taken into consideration to construct interpretative models. The term “*grounded*” refers to a theory that emerges from the ground, that is, from below. With this methodology, it is possible to build a rooted theory in the text under examination, which in the case of the research presented here coincides with the transcribed interviews. The objective of this methodological process is to construct a psycho-social reality from the point of view of the participants, taking as an object of analysis what they have affirmed (symbolized through language) [22].

Specifically, the procedure provides three phases of data coding: open coding, axial coding, and selective coding.

## 5. Results

The data analysis produced (*n* = 20) 20 categories (listed in Table 2) grouped into 6 (*n* = 6) macro-categories: (1) *psychophysical characteristics*; (2) *learnings*; (3) *relational aspects*; (4) *collaborations*; (5) *extracurricular activities*; and (6) *teacher training.*

In the first phase of the analysis, the qualitative data were codified through the coding method defined as “open” to indicate the action of opening the text and letting ideas emerge from it. This analysis led to the attribution of 408 codes to the concepts that emerged from the interviews. Two other phases of analysis then followed: in the first phase, these codes were grouped into 20 categories, and in the second, 6 macro-categories were identified. They are explained in Table 2 below. Finally, the literature review was taken into consideration in order to make comparisons and build an interpretative structure that took into account linguistic expressions and the interpretative models used within the psychological sciences and the studies on collusive arrangements [23]

### 5.1. Theme 1: Psychophysical Characteristics of Pupils

In the first macro-category identified, the teachers described aspects of the student relating to physical health and to the emotional–behavioral level, also making comparisons with other disabilities with which they came into contact during their working career. As far as health problems are concerned, the interviews show that those of a cardiac nature prevail and, in some cases, even heart surgery was necessary. In particular, two individuals received heart surgery at birth and another during primary school years. Another support teacher (n.4) states: “*she was absent for a long time because she underwent heart surgery, due to heart problems from which she suffered*” (n.4).

Other problems associated with DS are hematological, renal, and bronchial. Support teacher n.3 recalled a student suffering from bronchial issues, claiming “*she was in intensive care*” and therefore “*for a long time she was absent from school*” (n.3).

Another teacher expressed: “*my pupil suffers from severe dermatitis with flaking and therefore sometimes we have to intervene to put plasters on*” (n.15), and teacher n.9 claimed that “*when she does physical activity she gets tired because of her heart problems […]*” and her being “*[…] overweight […]*” (n.9).

As far as the emotional–behavioral level is concerned, some peculiarities emerge which are repeated in several descriptions. In particular, several teachers speak of students who are “*very affectionate both with her classmates and with the teachers*” (n.6). Furthermore, they claim that “*she seeks physical contact with everyone*” (n.7), thus establishing a relationship between teacher and pupil. However, it seems that most individuals with DS do not always manifest themselves in this way right away, considering that at the beginning, in most cases, the pupil “*was very closed, she even turned her back on me*” (n.12), and “*he tended to always assume an oppositional attitude towards me because he had little confidence in me*” (n.6), as well as the pupil supported by teacher n.11, who states: “*the first months were characterized by refusals, crises of opposition and scarce collaboration.*” These oppositional attitudes, in some cases, are also manifested in other specific contexts, as well as in the first phase of knowledge. In particular, “*it was never necessary to intervene in a very abrupt way otherwise the wall would be raised. Dramatically imposing an activity implied its refusal*” (n.11). Among the weak points of the pupil described by teacher n.14 is his “*difficulty in not accepting a reprimand or an imposition*.”

A similar affirmation is typical of teacher n.10, who also supported a pupil with behavioral problems and, in comparing them, she states: “*a characteristic of the other child I follow is the character malleability. Instead, the biggest stumbling block of the child with DS is the very tough temperament, sometimes he manages to manipulate you easily.*”

In addition to stubbornness, another element shared by various teachers’ descriptions of their pupils with DS concerns the desire to “*be noticed and acclaimed by others because they like being the center of attention*” (n.10). This also applies to the pupil supported by teacher n.9: “*when she finishes a task, she goes to her colleague at the desk and receives applause because she is a pupil who seeks consensus*” (n.9). Furthermore, in several interviews teachers claimed they had to encourage the pupil to participate “in group life” because they did do so of their own free will. An example is the child followed by teacher n. 11, which “*by itself did not initiate anything, there was a constant need for support and mediation. This year I have a very serious case and that is a non-verbal autistic. Paradoxically, he takes more autonomous initiatives than last year’s child with DS*” (n.11).

However, from the various descriptions it emerges that this difficulty is also present in some children with DS, so much so that the pupil followed by teacher n.11 “*was not always able to express his needs affective with explicit requests*” (n.11). Furthermore, there are those who are unable to communicate their own needs, such as going to the bathroom. Teacher n.6 underlines: “*One of his difficulties is instead that of being able to communicate his emotions and his needs, especially those of a physiological nature*” (n.6). For this reason, it is the teachers themselves who, in this case, must remind the child to go out to the bathroom.

In general, although the picture is heterogeneous, the difficulty in acquiring autonomy is highlighted by several teachers.

Additionally, some support teachers made comparisons between DS and other disabilities they had witnessed in their career. In particular, they mainly referred to a child with autism spectrum disorder that they had in their care, focusing mainly on the affective, behavioral, and language-related areas. Teacher n.7 compared her student with DS with a previous student who had autism spectrum disorder and claimed: “*If the first one really wants compliments and hugs, he instead was very detached, he didn’t even want to shake my hand*” (n.7).

### 5.2. Theme 2: Learnings

The linguistic competence was very compromised in most cases. Specifically, the major difficulties were highlighted at the phonological level. In fact, interviewee n.4, when speaking of her student with Down syndrome, stated: *“She showed various difficulties in the linguistic area, she spoke little and when she tried to do so, it was difficult to understand her because she could not pronounce many sounds and words”* (n.4). This was based on the fact that “*it is hard to understand”* (n.1) and on the “*problems with articulation”* stated by teacher n.7, and there were frequent anomalies in the auditory and oral structure. Teacher n.3 commented: *“The girl has a lot of difficulty pronouncing some sounds and words, due to some somatic traits typical of Down syndrome, including a large tongue and small mouth”* (n.3). Thanks to targeted interventions, improvements are possible in this area. Teacher n.2 states: *“However, also thanks to the work carried out in collaboration with the center she attends, she has made numerous progresses, above all from a linguistic point of view”* (n.2).

Although verbal production is compromised in many individuals with DS, some teachers claim that they are still able to understand the pupils because they resort to gestural means of communication, such as the child supported by teacher n.7: “*he manages to communicate everything with non-verbal communication”* (n.7).

To a lesser extent, many other pupils with DS supported by the teachers interviewed also showed various problems in the logical–mathematical field. This is demonstrated by comments such as: “*He has difficulty in the logical-mathematical area”* (n.13), or such as: *“Another compromised area is that of abstract thinking, in fact, especially for mathematics he has always need to have concrete references”* (n.3). The need to make abstract concepts concrete is underlined by various teachers, many of whom, especially for learning mathematics, use a lot of concrete material. In addition to the difficulties encountered in linguistic and logical–mathematical areas, almost all interviewees highlighted others at the motor level, saying, for example, that the *“motor-practical”* area is among the very compromised areas (n.11), regarding difficulty with coordination and dynamic balance. In this regard, teacher n.5 states: “*sometimes she is a bit clumsy, especially in managing her balance, because she tends to fall into certain stereotypes”* (n.5)*;* moreover, in the case of the pupil supported by teacher n.3, it was also necessary to use orthopedic aids: *“she has problems walking and therefore wears braces”* (n.3). The “*somewhat awkward posture”* (n.6) and difficulties in *“global coordination”* (n.12) are highlighted by various teachers; however, the element that most unites almost all stories concerns the presence of *“difficulties with the fine-motor coordination that he shows above all in the writing process”* (n.2) or when the student has to *“cut even small pieces of paper”* (n.6). In light of these difficulties, several interviewees underline the need for specific tools and targeted interventions, when, for example, they say: *“We are working a lot on pen/pencil grip”* (n.7) and *“the little girl used the handle and, thanks to it, she was able to obtain improvements”* (n.4*)* in the writing process. Furthermore, one teacher deemed it necessary to intervene with total physical guidance of the movement of the child’s hand: *“To work on fine motor skills, on the correct grip and pressure to apply on the pen, I had the need to work a lot with prompting, therefore in close contact with him”* (n.11). Similarly, teacher n.13 claimed that they *“had to accompany him with their hand” (n.13)*. In fact, prompting is considered a very suitable teaching technique when working with children with intellectual disabilities, especially if these are severe and profound [17]. Overall, there is an evident need to materialize mathematical concepts and to create playful environments, as teacher n.10 explains: “*We have come to associate number with physical quantity and to count. We proposed several times to the whole class to play bingo and the child with DS did the bingo, fishing and saying the numbers. Other times we all play together with the game of the goose or with the sponge dice.”*

Another useful element for children with intellectual disabilities and which several interviewees also talked about is *“the visual agenda that scans the moments of the day”* (n.8) which allows the student to *“orient himself in time and understand the lessons of the day”* (n.14). Thanks to visual agendas, the children are able to understand which lesson they will have in a given hour and, therefore, which notebook they will have to use. Interviewee n.8, like others, explains that the student “*uses the diary so as to be able to identify the exercise book to take. Each notebook is of a different color and each color represents a discipline”* (n.8).

### 5.3. Theme 3: Relational Aspects

This macro-category includes categories relating to the type of relationship established between the pupil with Down syndrome and the teachers and that established with classmates.

As far as the relationship between the student with disabilities and the teacher is concerned, a fairly homogeneous picture emerges, as in all cases it is described by the teachers in positive terms. Differences are identified only in relation to the time taken to create a relationship of this type. In some cases, a strong bond was created between the teacher and the child from the beginning, and this is demonstrated in the sentiments of teacher n.8: “*We met in September and immediately established a solid relationship. He trusts me and I am his point of reference”* (n.8), and those of teacher n.13: *“harmony and synergy was created with me right away”* (n.13). Positive relationships also emerge from the other stories, but for some it took longer for them to be created. An example is that of interviewee n.6, who states: *“Initially it was a bit difficult to establish a relationship with her then over time, after a few months, I managed to get accepted. Now the relationship is much better. It is a relationship of empathy and affection between me and her”* (n.6). A similar description is given by teacher n.5: *“during the first months of school she was sitting with her back to her […] we had no response from her, not even with gestures. Subsequently, little by little she showed herself more available and the relationship between us undoubtedly improved”* (n.5).

The relationship created with classmates is also positive. In fact, some teachers state: “*he has established a good relationship with his classmates*” (n.14) and “*He is fully integrated into the class group, his classmates involve him in every activity, they are supporters of his growth path, in fact, they always help him in the learning processes and for the discourse linked to autonomy*” (n.8).

The other pupils are therefore real resources for the child with disabilities, as explained by teacher n.12: *“The class group was fundamental. It is a class that has easily welcomed the little girl, who joke, play and have fun with her.”*

However, not all teachers interviewed were satisfied with the work carried out for the purpose of promoting inclusion, above all due to the anti-contagion restrictions and particular behaviors of colleagues, as stated by interviewee n.12: *“The problem of Inclusion in this class involves teachers who complain that the child and I are annoying others and therefore invite us to stay at the back of the class. Unfortunately, this somewhat limits the process of inclusion”* (n.12).

Another criticism is made explicit by teacher n.3, who, after having stated: “*The relationship with classmates is positive*” and that they “*are affectionate and inclusive*”, then points out: “*I however, there are specific inclusive interventions proposed by the teachers, in the sense that the girl carries out work separated from the rest of the class-group and sometimes even outside the classroom when it is not possible to work in class because there are situations in which teachers wish to have absolute silence in the classroom*” (n.3). Furthermore, many teachers apply collaborative strategies in order to facilitate curricular learning, enhance self-esteem, social skills, and promote inclusive processes within the class group. Teacher n.4 states: “*We often used a peer-to-peer strategy. Many jobs were carried out in small groups, in each of which a child occupied the role of tutor and therefore had the task of helping the other classmates. In this way, the girl felt more accepted and included by her classmates*” (n.4).

### 5.4. Theme 4: Collaborations

The fourth macro-category includes aspects relating to programming, the type of relationship established between colleagues, between teachers and family, and between health specialists. These elements and the coordination between the various figures that come into play are of fundamental importance for the promotion of a truly inclusive school.

The panorama relating to the teaching team is not entirely homogeneous because many support teachers stated that *“the colleagues are very collaborative”* (n.10) and that “*the learning area is tackled thanks to teamwork with all the other colleagues in the class*” (n.1), but there are others who are dissatisfied with the type of relationship established with the colleagues, as the activities carried out are not always shared. Specifically, teacher n.3 stated: *“The girl could perform certain exercises, but she should be constantly involved and my colleagues lack this”* (n.3) and another teacher explained how sometimes she received *“the request by some colleagues to leave the classroom with the disabled child”* (n.4). Even during the planning phase, several interviewees point out that *“there is no collaboration and comparison”* (n.15) within the team, and for this reason there are those who claim to have *“drafted the Individualized Education Program by herself without collaborating with the other teachers”* (n.8).

However, according to the teachers, their decisions are not always shared by the family, even with reference to the individual education program (IEP). For example, the father of a child, contrary to the teacher’s choices, *“wanted his son to follow the class schedule for all disciplines”* (n.14). Another teacher, speaking of the mother of the child she is in charge of, said: “*she doesn’t trust us. She does not listen to our advice, on the contrary it is she who tells us what we must do”* (n.12). In fact, in some cases parents appear to be too demanding, even going so far as to give explicit indications to teachers. Confirming this are the words of teacher n.5: *“Parents are very present, but sometimes they come to give you directives on how you have to work at school to try to follow the work done at home”* (n.5). Teacher n.4 also claims: *“the mother necessarily wanted her daughter to do everything her classmates did, even when it was not possible to treat her in the same way as the others”* (n.4). Teachers believe that the reason for these two attitudes, and that of other parents, derives from the fact that the family “*has very high, unbalanced expectations, as if it wanted to go beyond its limits. In fact, sometimes his mother asked me to assign him more tasks”* (n.11).

Attitudes of this type, implemented by the family, could have repercussions on the child. This was the case with the child supported by teacher n.11 who showed a lot of performance anxiety which, according to the teacher, *“probably derives from the pressure of the mother who has very high expectations”* (n.11).

Despite the strong desire for the child to achieve uncalibrated goals, interviewees suggested that almost all families were aware of the child’s skills. Several teachers, speaking of the pupil’s family, confirmed that they were *“very aware of the girl’s strengths and weaknesses”* (n.3).

Overall, in most cases the confrontation between the family and the teachers was constant, yet there was also collaboration. A different situation, however, emerges with reference to the relationship between the school and the specialists who support the child. In fact, there were few cases in which teachers state that *“a network has been created between operators, school and family”* (n.9) and that *“the dialogue with the therapist is constant”* (n.1). From the speeches of the interviewees concerning the GLO meetings, it emerges that in several cases *“the figures outside the school who look after the child were not present”* (n.14).

### 5.5. Theme 5: Extracurricular Activities

Among the extracurricular activities carried out by children with DS supported by the support teachers interviewed, it appears that those of a sporting and therapeutic type prevail. Specifically, the most practiced sports are swimming and dance, even if the latter is attended only by females. In addition to these two sports others are also practiced, but there is great variability. For example, teacher n.13 states: *“he plays football and swims to try to improve his coordination above all”* (n.13), and teacher n.12 claims that “*before Covid he used to dance, an activity that she likes it a lot”* but on some afternoons she is busy because *“she is followed by a speech therapist”* (n.12). Similar to this last child, many others attend speech therapy courses considering their linguistic difficulties. Furthermore, the therapeutic path of some also includes psychomotricity due to motor problems Only a few children attend activities aimed at developing socialization skills, despite "greater contact with their peers also in other contexts, would allow them to learn new relational competences" (n.6). Another fundamental objective to aim for according to some teachers concerns the acquisition of greater autonomy, above all in a perspective of future life. Teacher n.5 states: “*With the child with Down syndrome there is a need to work a lot on affectivity, on relationships and get them used to autonomy also for the future of society”* (n.5). Teacher n.15 also projects herself beyond the years spent within the school context and believes “*it is necessary to work on autonomy and communication because it is necessary to prepare it in view of a future life project”* (n.15).

Overall, it emerges that only some reflections on what could be implemented to improve the overall life of the pupil with DS are shared by several teachers. In particular, they are those in reference to organizing laboratory activities, to work on the area of language and autonomy.

### 5.6. Theme 6: Training of Teachers

On the subject of training, the picture is very homogeneous. The interviewees share a lack of specific and targeted training on the syndrome of the pupil they are in charge of, and none of the support teachers interviewed stated that they had followed any specific training on DS. Teacher n.11, for example, clearly states: “*Neither the school supported us, nor I have ever participated in specific courses”* (n.11). This emotion was shared by several others.

One teacher points out that “*we need more information on this syndrome, because we only have information from a clinical point of view.*” Furthermore, another teacher truly believes that “*at least one compulsory course is necessary for teachers who follow children with DS*” (n.10). This request is also typical of many other teachers who explicitly asked for greater training to improve the teaching process aimed at pupils with DS. Always reflecting on the training aspect, another teacher interviewed (n.3) points out: *“we should focus a lot on the training of teachers who are already in school and not only, as usually happens, on those who have yet to enter”* (n.3).

Many of the teachers interviewed in relation to training referred to the knowledge acquired during their university course and to the specialization course for support, saying: *“the school has not activated any courses. For me it is all the result of the degree and the TFA”* (n.8), as well as *“[…] the school has never offered me specific courses on DS, however, I am attending the TFA and it is helping me a lot because it gives a general picture of disability”* (n.6). Furthermore, most of the teachers spoke of independent training *“above all through research, reading and discussions with colleagues”* (n.7), and claimed that they documented themselves *“by buying and studying books”* (n.12) and that *“it is all the fruit of my common sense which led me to inform myself”* (n.10).

Other elements which were considered fundamental by several teachers for the purposes of their work with the pupil with DS concerned personal experience, comparison with colleagues, and observation. Overall, the teachers’ words show the lack of support from the school towards them.

## 6. Discussion

The study was conducted to detect the peculiarities of the didactic–educational and emotional relationship between support teachers and pupils with DS in primary school. The results illustrated below suggest interesting information about the peculiarities of the didactic and relational learning processes of students with DS and the coping strategies that the support teachers implement to address the critical issues that this specific disability entails.

With regard to the first theme (*Psychophysical characteristics of pupils*), which considered the limits in socialization and learning processes of pupils, the study underlines that the linguistic area is most compromised in DS students, in both written and oral production. This fact, states [4], is documented by numerous experimental studies [4], and difficulties in abstraction and fine motor coordination have also been identified by many studies. To answer the first difficulty, it appears that many teachers present concrete material and try to create situations that are very close to reality, especially to deal with mathematical concepts. In referencing Piaget’s stadial theory, we could say that we stop at the concrete operational stage, without reaching the formal operational stage [21].

Instead, regarding the difficulties relating to fine-motor coordination, from what the teachers say, they are visible above all in the writing process. In fact, several studies show that in subjects with Down syndrome the fine-motor components, as well as the gross-motor ones, occur much later than in typically developing peers. Specifically, it appears that if children with typical development learn to copy letters of their name between 36 and 48 months of age, children with DS acquire this skill between 108 and 144 months of age [4]. In addition to the need to make abstract concepts concrete, many teachers also underline the value of using many images, as it seems that they are effective for the learning process of the pupils with DS and their orientation over time. This could be due to the fact that visuo-spatial abilities are preserved in subjects with DS, as stated by Davis [6]. Another aspect underlined by Davis [6] and also emerging from this research, again with reference to children with DS, concerns their being less inclined to start a game than their peers. For this reason, some teachers highlight the need to encourage the student to participate in various activities, otherwise he/she will not do so. In fact, according to many teachers interviewed, pupils with Down syndrome in their care need to spend more time with their peers in extracurricular environments to be able to acquire social skills. This need confirms the mistake often made in generalizing and stating that people with DS are sociable. This feature could occur in some individuals, but it is not a trait attributable to the syndrome itself [25,26,27].

A further need that the participants highlighted concerns the need for specific training on Down syndrome, especially in relation to the didactic aspects. They state that they have never attended courses focused on this syndrome, but only on disability in general and on some disabilities specifically (such as autism spectrum disorder). Furthermore, with the exception of a couple of cases, all other teachers report the absence of support from the school for the management of pupils with DS. This negatively affects the inclusive process. Having more information on the learning characteristics of children with DS also means being able to improve the inclusion of these pupils [28,29,30].

In addition to the training aspect, teachers who participated in the study by McFadden [29] stated that they were able to better include the student with DS within the class thanks to the collaborative support received from colleagues. However, referring to the teachers interviewed, this last aspect was found to be absent in several cases. In several situations, the support teacher did not receive collaborative support from the class teachers. Other critical issues highlighted by various teachers were linked to the impossibility of applying some collaborative strategies, the absence of specialists during intervention planning meetings, and the families’ high expectations [30,31].

At this juncture, it is not possible to discuss the development path and of the pupils or to rely on taking charge of aspects concerning the pupils’ health and learning. The individualized education program should be co-constructed and shared between the school, the services that come into play, and the family [32], whose expectations unfortunately sometimes appear too high, idealizing the recovery processes of their children and devaluing the abilities of the teachers.

## 7. Conclusions

The purpose of the study was to provide an overview of DS at primary school level, placing emphasis on the teacher–pupil relationship in support of the learning and relational processes of pupils with this disability.

The support teachers interviewed highlight the importance and the need to fully understand the characteristics and peculiarities of this specific disability to have specific information on the syndromic picture and on the factors that could affect learning and inclusion processes. For example, being aware of the fact that individuals with DS generally have good visual-spatial skills suggests that the teacher should intensify the use of visual aids during lessons. In addition to the limited training of teachers on the specific disability referred to, other critical issues emerge from the study which in some way affect the process of learning and inclusion of students with DS. Specifically, the critical points that emerged in several cases concern the lack of collaboration between class and support teachers and the absence of health specialists in the school context. These two elements, together with the climate present in the classroom, outline the type of educational context formed. The latter does not only concern the physical environment, but also the relationships that are created between the different figures (internal and external to the school).

The scarce collaboration, in some cases between support teachers and class teachers, and in others between teachers and clinicians, implies the inability to set up a psychoeducational intervention from a co-constructive perspective. Often families are left alone and children with Down syndrome are trapped in the transition to adulthood in a subsidiary social system, especially in the south [31].

The results suggest that the process aimed at achieving more solid inclusion of pupils and tailored psychological training [32,33,34] with DS in Italian schools, and in general with other disabilities and diversities [35] is ongoing.

## 8. Limits

This study certainly has some important limitations. The first concerns the limited number of participants; in the absence of an ad hoc register, identifying teachers who have assisted and who are assisting students with DS was only possible through a ball sampling system starting from the knowledge of the participants. In the future, it would be desirable to identify other support teachers through the associations of people and parents with DS. Another limitation concerns the fact that students with DS or other care givers were not directly involved in this study. A third limit concerns the fact that the qualitative nature of the research allowed a contextualization of the results to the Italian context and to the structure and mission of the Italian school; the results and the considerations have been produced taking into account these socio-cultural dimensions, difficult to find in other realities.

## Figures and Tables

**Table 1:** Description of teachers’ characteristics (*n* = 15).

	Gender	Age	Years of Experience as a Curriculum Teacher	Years of Experience as a Support Teacher	Education	Diploma as a Special Education Teacher (TFA)	Contract	School Location
1	F	42	0	20	Degree in Primary Education	yes	permanent	Bari
2	F	44	1	2	Degree in Primary Education	yes	temporary	Benevento
3	F	26	1	1	Degree in Primary Education	in progress	temporary	Roma
4	F	43	8	9	Degree in Primary Education	yes	permanent	Napoli
5	F	50	0	25	Teaching diploma	yes	permanent	Sorrento
6	F	25	0	1	Degree in Primary Education	in progress	temporary	Roma
7	F	40	0	14	Degree in Primary Education	yes	permanent	Campobasso
8	F	27	2	1	Degree in Primary Education	yes	permanent	Milano
9	F	54	0	22	Degree in Primary Education	yes	permanent	Pescara
10	F	30	1	1	Degree in Primary Education	no	apprenticeship	Vicenza
12	F	45	3	10	Degree in Primary Education	yes	permanent	Napoli
12	F	46	0	19	Teaching diploma	yes	permanent	Napoli
13	F	38	0	2	Degree in Primary Education	yes	temporary	Foggia
14	F	51	0	30	Teaching diploma	yes	permanent	Caserta
15	F	45	18	5	Teaching diploma	yes	temporary	Napoli

**Table 2:** Macro-categories and sub-categories.

Macro-Categories	Sub-Categories
1. Psychophysical characteristics of pupils	Health problems. Emotional–behavioral aspects. Comparison with other disabilities.
2. Learnings	Compromised areas and various difficulties. Related characteristics and preferences to learning. Strategies and teaching methods. Teaching materials. Schoolwork.
3. Relational aspects	Pupil/teacher ratio. Relationship with classmates and class climate. Collaborative strategies and inclusion.
4. Collaborations	Program. Relationship between colleagues. Teacher–family relationship. Relationship between school, services and family.
5. Extracurricular activities	Homework. Extracurricular activities. Teacher reflections.
6. Teacher training	Teacher training. School support.

## Data Availability

Data are available upon request from the first author.

## Appendix A

Interview guide
***Psycho-physical characteristics of pupil*** (1) Would you describe the student with Down syndrome that you follow or have followed, explaining the gender, age, school, and class attended? (2) In your opinion, on an emotional level, what are the strengths and weaknesses that you manifested? (3) In your opinion, which are the most compromised areas? (4) Have any clinical complications occurred during the lessons? (health problems such as tachycardia, etc.) ***Relational aspects and inclusion*** (5) Could you tell me about your experience with this student? How long have you been following him? What kind of relationship did you establish with him/her? (6) In your opinion, is/was the student included within the class group? If not, why? What problems emerge/were emerging in relation to inclusion? ***Methodological decision-making dimension*** (7) How was the area related to the learning of the various disciplines or how was it addressed? (8) What strategies do/did you use? What others, in your opinion, could be used? (9) How are/were the learning assessments carried out? (the student is/was alone or together with the other members of the class) (10) Is/was a differentiated programming drawn up? ***Extracurricular context*** (11) Is/was the pupil with Down syndrome given homework? Did they follow you home? Do you do homework with someone? (12) Did the child carry out extracurricular activities? If yes, with whom? If not, do you think they would be important? With what aims and objectives? ***Teacher’s experiences and reflections*** (13) How did you learn to work with pupils with Down syndrome? Did you take specific courses? Did the school support you? (14) Have you also had experience with students with other disabilities? If so, what do you think is the major difference between people with Down syndrome and people with other disabilities? (15) In your opinion, is the family aware of the strengths and weaknesses of the child? (16) What do you think could be done to improve the situation of this child with Down syndrome? ***Relationship between school and family*** (17) Does the operational working group meet periodically? If not, why? (18) Are/were you able to collaborate with the family of the pupil with Down syndrome? If not, why? If yes, in what?
